# Genome-Wide Identification of Trehalose-6-phosphate Synthase (TPS) Gene Family Reveals the Potential Role in Carbohydrate Metabolism in Peach

**DOI:** 10.3390/genes15010039

**Published:** 2023-12-26

**Authors:** Shihao Fan, Zhe Wang, Yuansong Xiao, Jiahui Liang, Shilong Zhao, Yihua Liu, Futian Peng, Jian Guo

**Affiliations:** 1 College of Horticulture Science and Engineering, Shandong Agricultural University, Tai’an 271018, China; 17866709337@163.com; 2 College of Agriculture and Forestry Sciences, Linyi University, Linyi 276000, China; wzhelaiwu@163.com (Z.W.); ysxiao@sdau.edu.cn (Y.X.); liangljh@163.com (J.L.); zslintaian@126.com (S.Z.); liuyihua@lyu.edu.cn (Y.L.)

**Keywords:** peach, TPS, T6P, gene family

## Abstract

Trehalose-6-phosphate synthase (TPS) is essential for plant growth and development, linking trehalose-6-phosphate (T6P) to carbon metabolism. However, little is known about the *TPS* gene family in peaches and their potential roles in regulating carbohydrates in peach fruit. In this study, nine *TPS* genes were identified in the peach genome and named according to the homologous genes in *Arabidopsis*. Phylogenetic analysis showed that three subfamilies were identified, including TPSI, TPSII-1, and TPSII-2, which were also consistent with gene structure analysis. Considerable cis-elements were enriched in the promoters, including plant hormone-related elements. Tissue-specific analysis showed that these *TPS* genes were mainly expressed in leaves, stems, and fruit, showing different expression patterns for each gene. In addition, during fruit development, the content of trehalose-6-phosphate (T6P) was positively correlated with the expression of *PpTPS7a* and negatively with sucrose non-fermenting-1-related kinase 1 (SnRK1) activity. Transient overexpression and silencing of *PpTPS7a* in peach fruit validated its function in regulating T6P content and SnRK1 activity.

## 1. Introduction

Trehalose (α-d-glucopyranosyl-1,1-α-d-glucopyranoside) is a non-reducing disaccharide in which two glucose units are linked by α, α-1,1-glycosidic bonds [1]. Most species, including bacteria, yeast, and plants, can use it as an energy and carbon source. Trehalose also contributes significantly to the processes of signal transduction, membrane protection, and osmotic pressure maintenance [2,3]. Trehalose functions as a signaling molecule in directing metabolic pathways, regulating plant development, and protecting proteins [4]. Under adverse environmental conditions, such as low temperatures, drought, and salt stress, trehalose is synthesized massively [5].

In the metabolism of trehalose, T6P is synthesized by TPS using glucose-6-phosphate (G6P) and UDP-glucose (UDPG) as substrates, while trehalose is synthesized by trehalose-6-phosphate phosphatase (TPP) using T6P as substrates. As a substrate, trehalose is used to synthesize glucose by trehalose hydrolase (TRE) [6,7]. TPS genes were found in many plant species [8]. For example, there were 11 *TPS* genes (*AtTPS1*–*AtTPS11*) in Arabidopsis, 11 members in rice (*OsTPS1*–*OsTPS11*), and 12 members in Populus (*PtTPS1*–*PtTPS12*) [9,10,11]. However, *TPS* genes in peaches have not been systematically investigated.

*TPS* genes have been found to regulate carbohydrate metabolism, which further affects plant growth [12]. *TPS* genes are further divided into two subfamilies, class I and class II. In *Arabidopsis*, *TPS* genes in class I (except *AtTPS3*) have TPS enzyme activity, while they are absent in class II [10,13]. *TPS* genes in class I could improve plant tolerance to stress. For example, overexpression of the *OsTPS1* gene increased tolerance to drought, high salinity, and cold stress in rice [14]. *AtTPS1* is crucial for the metabolic regulation of *Arabidopsis thaliana* [15]. Recently, the functions of *TPS* genes in class II have been discovered. *ATTPS5* functioned as a negative regulator of ABA signaling and regulated alginate content [16], while *ATTPS6* was involved in the regulation of plant architecture [17]. Overexpression of *OsTPS8* in rice improved plant salt tolerance [18]. Then Overexpression of the wheat trehalose 6-phosphate synthase 11 gene enhances cold tolerance in *A. thaliana* [19]. To better understand the function of *TPS* genes, it is crucial to further identify and characterize the *TPS* gene family in plants.

T6P plays a crucial role in plant growth and development and participates in trehalose biosynthesis [20]. T6P had been identified as inhibiting the activity of sucrose non-fermenting-1-related kinase 1 (SnRK1) [21], while changes in SnRK1 activity could also have an influence on the content of sucrose and T6P [22]. In our earlier studies, SnRK1 boosted sorbitol metabolism by activating sorbitol dehydrogenase (SDH), and it also increased sucrose buildup in peach fruit. SnRK1 is involved in sugar metabolism and has the potential to be used for improving fruit quality [23]. These studies showed a regulatory network among T6P, sugar, and SnRK1.

Peach (*Prunus persica* L.) is an economically important fruit crop in the world and is always considered the model plant in the Rosaceae family due to its small genome size [24]. During the past several years, a number of studies have reported the analyses of large-scale genome resequencing data in peach, with an attempt to understand the origin, evolution, and domestication of peach, as well as to identify genome regions highly associated with important traits [25]. However, little is known about the *TPS* gene family in peaches and their potential roles in regulating carbohydrates in peach fruit. In this study, we carried out genome-wide identification of *TPS* genes and analyzed their function in peach fruit. The identified *TPS* genes and their potential function in regulating SnRK1 provide insights into carbohydrate metabolism in peach fruit.

## 2. Materials and Methods

### 2.1. Identification of PpTPS Gene Family in Peach

The whole genome sequence of the peach cultivar ‘Lovell’ was downloadable at (*P. persica* genome assembly Prunus_persica_NCBIv2—NCBI—NLM (nih.gov)) (accessed on 11 August 2023) [26]. The conserved domain (PF00982) of the TPS protein was obtained from Pfam (xfam.org) (accessed on 15 August 2023). HMMER (biosequence analysis using profile hidden Markov models) was used to construct the HMM model to obtain the candidate TPS proteins in peach. The identified *TPS* genes were further deposited into NCBI to check the TPS domain. The chromosome length was extracted from the gene annotation file. As well, genomic locations of *TPS* gene family members on the peach genome were extracted, and gene distribution was plotted using ‘Gene Location Visualize (Advanced)’ in TBtools ((Toolbox for Biologists) v1.120) [27]. The physical and chemical properties of PpTPS proteins were further analyzed using TBtools [28].

### 2.2. Collinearity Analysis and System Evolution Analysis

The amino acid sequences of *TPS* genes in tomato (*Solanum lycopersicum*), apple (*Malus domestica*), and Arabidopsis (*A. thaliana TAIR10*) were downloaded from the NCBI (www.ncbi.nlm.nih.gov) website (accessed on 17 August 2023). Multiple sequence alignments were carried out using clustalW (genome.jp) (accessed on 17 August 2023) [29]. A neighbor-joining tree was constructed with the default parameter using MEGA (MEGA11) software [30].

### 2.3. Analysis of Gene Structure and Conserved Domain

MEME5.5.3 (meme-suite.org) (accessed on 18 August 2023) was used to predict and assess the conserved protein motifs of the PpTPS protein sequence. To produce an XML file, the input motif parameter is set to 15, while the other parameters are left at their default values [31]. To create the structural diagram of the conserved protein motif in TBtools’ gene structural view (Advanced). The peach genome GFF3 format file was entered in order to determine the CDS and UTR structure of the displayed gene.

### 2.4. Promoter Element Analysis

From the JGI website (Phytozome (doe.gov)) (accessed on 18 August 2023), the promoter sequences of *PpTPS* genes were selected and downloaded as 1.5 kb upstream 5′UTR. To predict the promoter elements, all promoter sequences were submitted to the PlantCARE database. The distribution and heat map of the promoter elements were plotted using TBtools [32].

### 2.5. Plant Material Acquisition, RNA Extraction and qRT-PCR

The peach variety “Xiahui 5” is planted in the experimental base of Shandong Agricultural University, with fruit round, creamy yellow pericarp, more than 80% of the fruit surface with red, white meat, delicate flesh, ripe in mid-July, and a fruiting time of about 100 days [33]. Samples of leaves, stems, flowers, and fruits were collected. Total RNA was extracted from the samples using an RNA extraction kit (Tiangen, Beijing, China), and the RNA purity was determined using an ultra-micro UV analyzer to confirm that the OD260/OD230 ratio was between 1.8 and 2.1. First-strand cDNA was synthesized using the PrimeScript first-strand cDNA synthesis kit (Takara, Dalian, China). Real-time quantitative polymerase chain reaction (qRT-PCR) was performed on the ABI7500 system using the SYBR premix ExTaq (Takara, Dalian, China) with the following procedure: 95 °C for 5 min, followed by 45 cycles at 95 °C for 10 s, 58 °C for 10 s, and 72 °C for 20 s. Reaction volume is 25 μL (including UItraSYBR Mixture (CWBIO, Taizhou, China) 12.5 μL, primer-F 0.5 μL, primer-R 0.5 μL, ddH_2_O 10.5 μL, cDNA 1 μL), with three biological replicates per sample. The relative expression level was calculated by the 2^−ΔΔCT^ method [34]. Primers for qRT-PCR are listed in Table S1.

### 2.6. Determination of T6P Content and SnRK1 Activity

The T6P content and SnRK1 enzyme activity were determined using ELISA kits. The peach fruits of the experimental and control groups were ground into powder in liquid nitrogen. Then, PBS lysate at a 1:9 ratio was added, and the samples were fully lysed by vortex oscillation. Centrifuged at 10,000 rpm for 20 min to collect the supernatant. Then, the supernatant obtained for each sample was divided into three for T6P and enzyme activity detection. Firstly, Set standard wells and test sample wells on an enzyme-labeled plate. Then, dilution of samples with sample diluent (sample final dilution is 5-fold). Adding HRP reagent, incubating at 37 °C, and adding chromogen solution, evade the light preservation for 15 min at 37 °C. Data were obtained by reading with a microplate reader (CMax Plus, Molecular Devices, Shanghai, China). The concentration and absorbance readings of the standards are used to create a standard curve in Excel, and the readings of the other samples were carried over into the standard curve equation to calculate the final result. The whole experiment was repeated three times.

### 2.7. Transient Overexpression and Silencing in Peach Fruit

The CDS sequences were obtained from the NCBI website and used for full-length primer design. Then, the *PpTPS7a* sequence was amplified from a cDNA library. BamHI and EcoRI were selected as restriction sites for the PRI101-AN vector. The amplified CDS sequences were further cloned into the pRI101-AN vector. EcoRI and BamHI were selected as restriction sites for the TRV2 vector. Finding the fragment with the best silencing effect on the *PpTPS7a* gene can be achieved by (Welcome to pssRNAit: Designing Effective and Specific Plant RNAi siRNAs with Genome-wide Off-target Gene Assessment (zhaolab.org)) (accessed on 23 August 2023) [35]. The specific silencing primers of *PpTPS7a* were designed and cloned into the TRV2 vector, and TRV1 was used as an auxiliary. Recombinant plasmid was added to the *Agrobacterium* competent cells GV3101 and placed on ice for five minutes, in liquid nitrogen for five minutes, in a 37 °C metal bath for five minutes, and on ice for five minutes to transform into colonies. In transient overexpression experiments, pRI101-AN is an empty vector as the control, while in transient silencing, TRV2 is the empty vector + TRV1 is the control. For the transient overexpression assay, peach fruits were cut into 1–2 cm-thick pieces, immersed in resuspended MMA buffer, and the liquid was injected into the fruit with an evacuation device. The infested fruit was then placed in MS medium in the dark for 24 h before being put under normal conditions. Finally, the samples were collected and frozen at −80 °C for downstream analysis.

## 3. Results

### 3.1. Identification of PpTPS Gene Family in Peach

**Figure 1:**
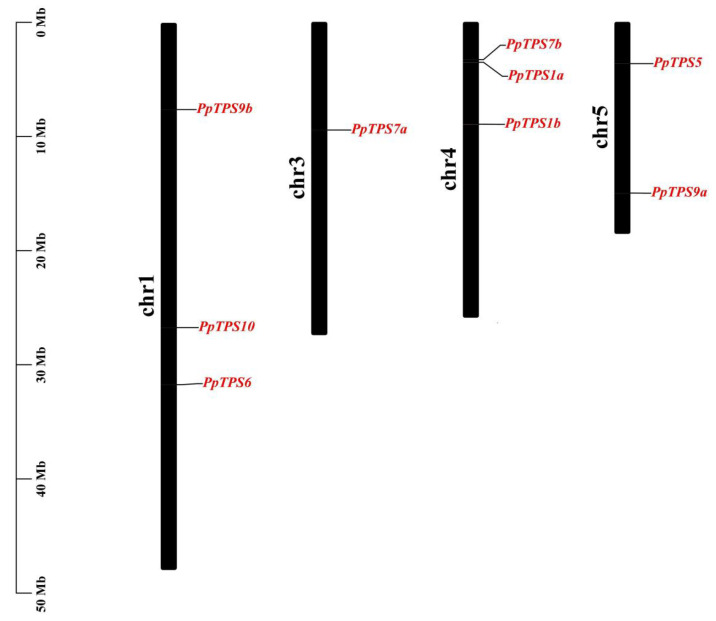
Distribution of *PpTPS* genes on peach genome. Chromosomes are indicated by long black rectangles. The *PpTPS* genes are marked in red.

To identify *TPS* genes, TPS and TPP domains were both considered using HMMER (v3.1). According to the *TPS* genes in *Arabidopsis* and tomato [36], nine *TPS* genes were identified in the peach genome. There were two TPS classes in *Arabidopsis*, including TPS1–4 in class I and TPS5–11 in class II [37]. Following the gene name in Arabidopsis, we named these nine *TPS* genes as *PpTPS1a*, *PpTPS1b*, *PpTPS5*, *PpTPS6*, *PpTPS7a*, *PpTPS7b*, *PpTPS9a*, *PpTPS9b*, and *PpTPS10*. *PpTPS* genes were irregularly distributed across four chromosomes. Among them, *PpTPS6*, *PpTPS9b*, and *PpTPS10* genes were distributed on Chr.1, *PpTPS7a* was distributed on Chr.3, *PpTPS1a*, *PpTPS1b*, and *PpTPS7b* were distributed on Chr.4, and *PpTPS1b* and *PpTPS7b* were found to be close to each other. *PpTPS5* and *PpTPS9a* are distributed on Chr.5 (Figure 1).

We investigated the physicochemical properties of these PpTPS proteins. As shown in Table 1, the predicted proteins of *PpTPS* genes include 840 (*PpTPS7a*) to 926 (*PpTPS1a*) amino acids, with an average of 871, and the molecular weight ranged from 95,686.72 Da (*PpTPS7a*) to 104,186.14 Da (*PpTPS1b*), with an average of 98,590.65 Da. The isoelectric point (pI) of a protein is an essential physiological measure that is mostly determined by the ratio of acidic to basic amino acids. The TPS protein has a theoretical isoelectric point between 5.4182 and 9.2656, and its isoelectric point is less than 7, indicating that it is an acidic protein. The hydrophilic coefficient of the protein is represented by the GRAVY value (Table 1). It is clear that *PpTPS9b* has the highest hydrophilicity, suggesting it is an acidic hydrophilic protein.

### 3.2. Gene Collinearity Analysis of PpTPS Genes

**Figure 2:**
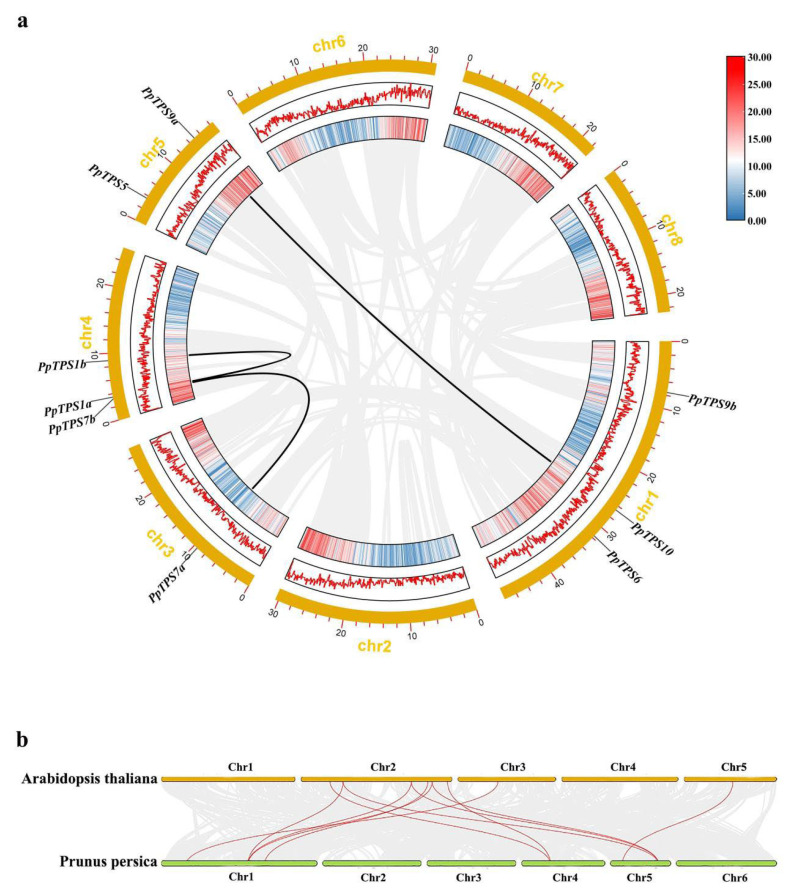
Collinearity analysis. (**a**) Collinearity analysis of *TPS* genes in peach. (**b**) Collinearity analysis of *TPS* genes in peach and *Arabidopsis*.

In order to investigate the evolutionary mechanism of the *PpTPS* gene, collinearity analysis was carried out, and three gene pairs, *PpTPS1a*/*PpTPS1b*, *PpTPS7a*/*PpTPS7b*, and *PpTPS9a*/*PpTPS10*, were identified (Figure 2a). This result indicated that the number of *PpTPS* gene families was mainly driven by duplication. In addition, we also constructed a collinearity map using the *PpTPS* genes in peach and Arabidopsis. A total of 11 pairs of genes were identified (Figure 2b), suggesting the high homology between these two species. The findings demonstrated that the *TPS* genes had high conservation during evolution.

### 3.3. Multiple Sequence Alignment and Phylogenetic Analysis

**Figure 3:**
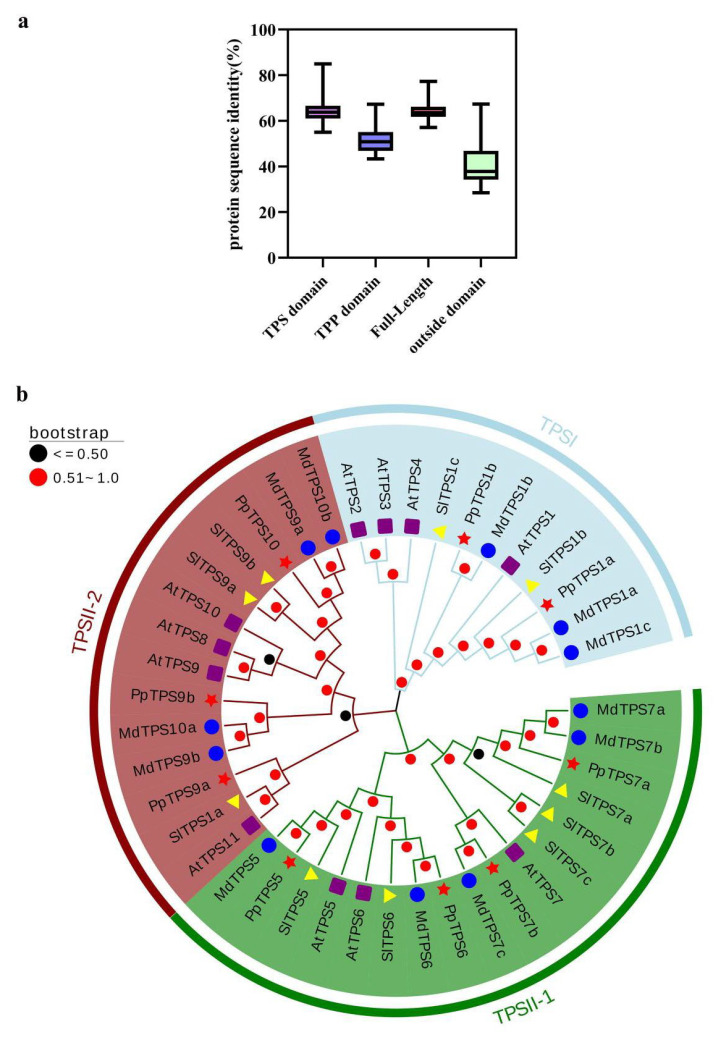
Multiple sequence alignment and phylogenetic analysis. (**a**) Calculation of sequence identity of TPS domain, TPP domain, full-length protein sequence, and extra-structural domain sequence. (**b**) Evolutionary tree constructed by *PpTPS* and *TPS* gene families of tomato, *Arabidopsis*, and apple. The yellow triangles represent tomatoes, purple squares represent *Arabidopsis*, blue circles represent apples, red pentagram represents the peach.

To evaluate the characteristics of the *PpTPS* gene family, we carried out multiple sequence alignments of amino acid sequences using Clustalw (2.0.11) tools (Figures S1 and S2). Further *PpTPS* gene sequences were put together to be compared (Figure S3). As a result, the catalytic core of *PpTPS* was substantially conserved, indicating its potential function in T6P synthesis. The homology of the nine *PpTPS* ranged from 57.14 to 77.26%, showing the highest homology between *PpTPS1a* and *PpTPS1b* and the lowest homology between *PpTPS5* and *PpTPS7a*. The average amino acid homology of the nine *PpTPS* was roughly 52%. Approximately 65% of the amino acid sequences in the TPS domain and 64% in the TPP domain showed homology. In comparison, only 40% of amino acid sequences were identical outside these two domains (Figure 3a).

Phylogenetic analysis was performed using *TPS* genes in tomato, apple, *Arabidopsis*, and peach (Figure 3b). The neighbor-joining (NJ) tree showed that there are two main subfamilies of *TPS* genes, TPSI and TPSII. In subfamily TPSII, two significant branches were observed and named (TPSII-1, TPSII-2) according to the bootstrap values.

### 3.4. Gene Structure and Protein Domain of PpTPS

**Figure 4:**
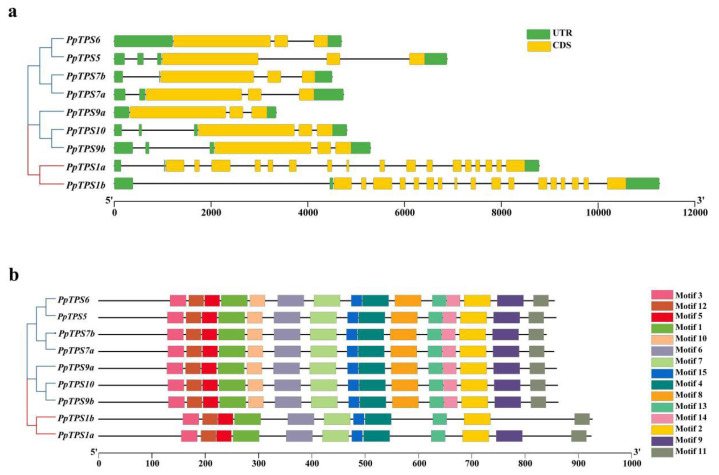
Gene structure and conserved motif analysis of *PpTPS* genes. (**a**) Gene structure of *PpTPS* gene. (**b**) Distribution of conserved motifs in *PpTPS* proteins.

The *PpTPS* gene structure was analyzed and viewed using TBtools software ((Toolbox for Biologists) v1.120) [27] (Figure 4a). The results showed that the number of introns and exons of *PpTPS1a* and *PpTPS1b* was higher than that of the other *PpTPS* genes. In addition, the CDS lengths of the nine *PpTPS* genes were highly similar (2523–2781 bp), although their total gene lengths varied greatly (3288–13,161 bp). Fifteen conserved motifs were identified in *PpTPS*, and their distribution in each subfamily was similar (Figure 4b), indicating similar functions as well. Motifs 1, 3, 4, 5, 6, 10, 12, and 15 together constitute the TPS domain, and Motifs 11, 13, and 14 constitute the TPP domain. All *PpTPS* genes have TPS and TPP domains. The analysis of conserved motifs and gene structure further supported the phylogenetic analysis of the *PpTPS* gene family.

### 3.5. Promoter Element Analysis

**Figure 5:**
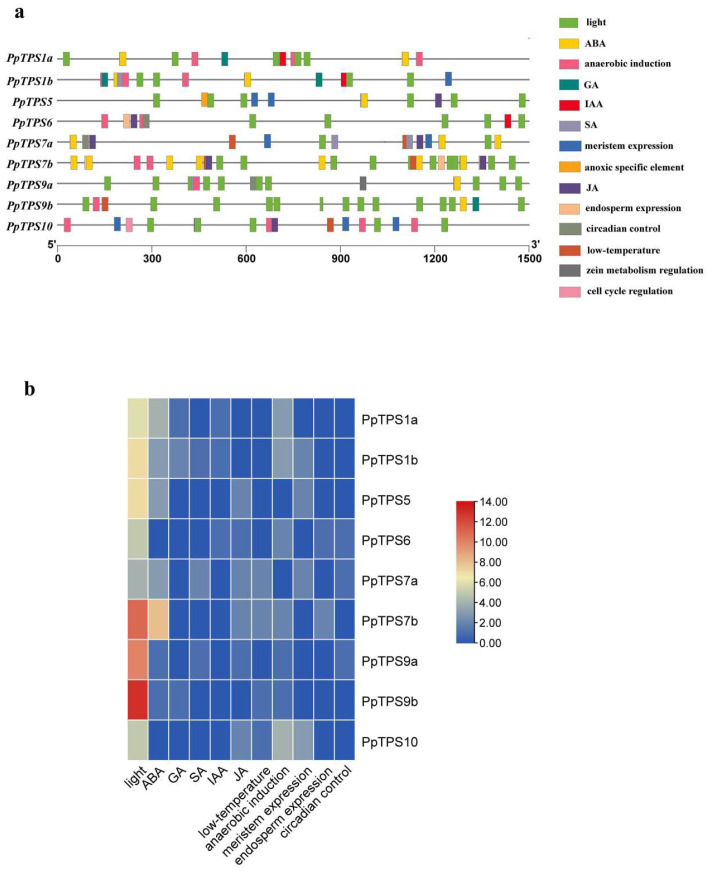
Analysis of the cis-elements of the *PpTPS* gene promoter. (**a**) Cis-elements identified in the promoters of the *PpTPS* gene. (**b**) Heat map of the cis-elements of each gene.

The promoter sequences of nine *PpTPS* genes (a 1.5-kb sequence upstream of the 5′UTR) were submitted to the PlantCARE database for promoter cis-element analysis, and a total of 21 cis-elements were identified. In addition to the basic promoter elements, such as TATA-box and CAAT-box, the remaining elements were related to plant hormones, plant growth and development, and abiotic stress (Figure 5a). Many plant hormone-related cis-elements were found in the promoter of *PpTPS7b*, while defense and stress-related cis-elements were found in *PpTPS1a* and *PpTPS9a*, and cis-elements that respond to low temperatures were found in *PpTPS7a*, *PpTPS7b*, *PpTPS9b*, and *PpTPS10* (Figure 5b). The presence of hormone response elements (ABA, GA, SA, and IAA) indicated that *PpTPS* genes might be involved in plant hormone signaling pathways, which was consistent with a previous study [16]. For example, *OsTPS8* controls yield-related traits and confers salt stress tolerance by enhancing suberin deposition. *TPS5* negatively regulates ABA signaling by influencing ABA, ROS level, and NR activity during seed germination and stomatal closure in Arabidopsis [18]. As well, the ABRE element in the *TPS* gene promoter can regulate the response of genes to ABA and salt stress [38,39].

### 3.6. Tissue Specific Expression Pattern of PpTPS Genes in Peach

**Figure 6:**
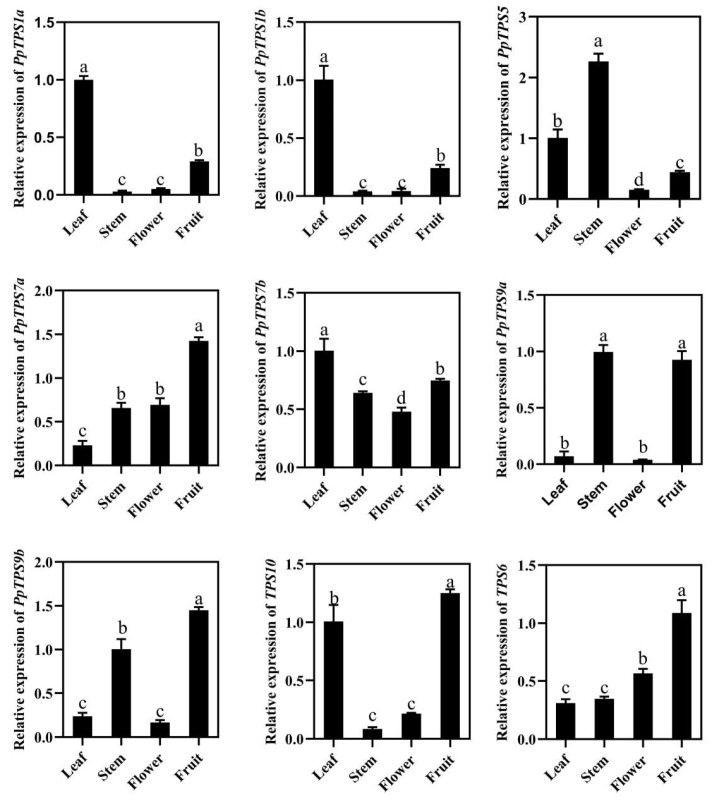
Tissue specific expression analysis of *PpTPS* genes. Note: Different letters indicate significant differences.

Tissue-specific expression patterns of *PpTPS* were analyzed. In general, the *PpTPS* gene is preferentially expressed in specific tissues. In subfamily TPSI, *PpTPS1a* and *PpTPS1b* were specifically expressed in leaves, showing similar expression patterns. In subfamily TPSII, *PpTPS5* was highly expressed in stems, *PpTPS6* and *PpTPS7a* were highly expressed in fruits, *PpTPS7b* was non-specific, *PpTPS9a* and *PpTPS9b* were highly expressed in stems and fruits, and *PpTPS10* was highly expressed in leaves and fruits (Figure 6). These results indicated that *PpTPS* genes might participate in plant and fruit development in different manners.

### 3.7. Expression Profile of PpTPS during Peach Fruit Development

**Figure 7:**
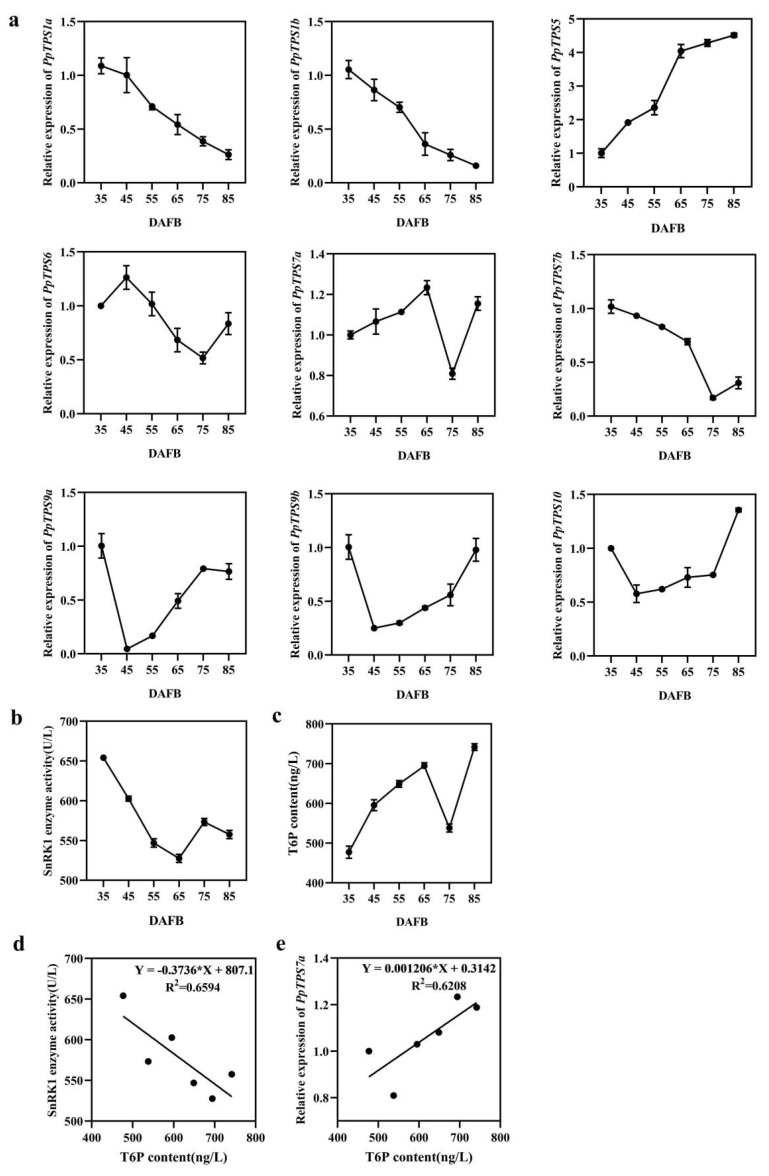
(**a**) Relative expression levels of *PpTPS* gene during fruit development. (**b**) Determination of T6P contents during fruit development. (**c**) SnRK1 activity during fruit development. (**d**) Correlation analysis between T6P content and SnRK1 activity. (**e**) Correlation analysis between *PpTPS7a* expression and T6P contents. Note: DAFB means number of days since flowering.

To explore the expression pattern of *PpTPS* genes during peach fruit development, their expressions were further analyzed. As shown in Figure 7a, the expression patterns of genes in subfamily TPSI were consistent and gradually decreased during fruit development. However, the gene expression patterns were different in subfamily TPSII. As we know, T6P was synthesized by TPS using glucose-6-phosphate (G6P) and UDP-glucose (UDPG), which further participate in carbohydrate metabolism via SnRK1. To understand the potential roles of *PpTPS* in fruit development, the T6P contents were determined in fruit at different developmental stages. The results showed that the T6P contents increased during fruit development while decreasing at 75 DAFB (Figure 7c). To identify the key *TPS* gene responsible for T6P synthesis, a correlation analysis between T6P and *TPS* gene expression levels was carried out, showing that *PpTPS7a* has the highest correlation coefficient (Figure S4 and Figure 7e). Due to the correlation between T6P and SnRK1 activity, we measured the SnRK1 enzyme activity at different stages of development stage (Figure 7b) and found that it was completely opposite to the change in T6P content (Figure 7d).

### 3.8. Functional Validation of PpTPS7a in Peach Fruit

**Figure 8:**
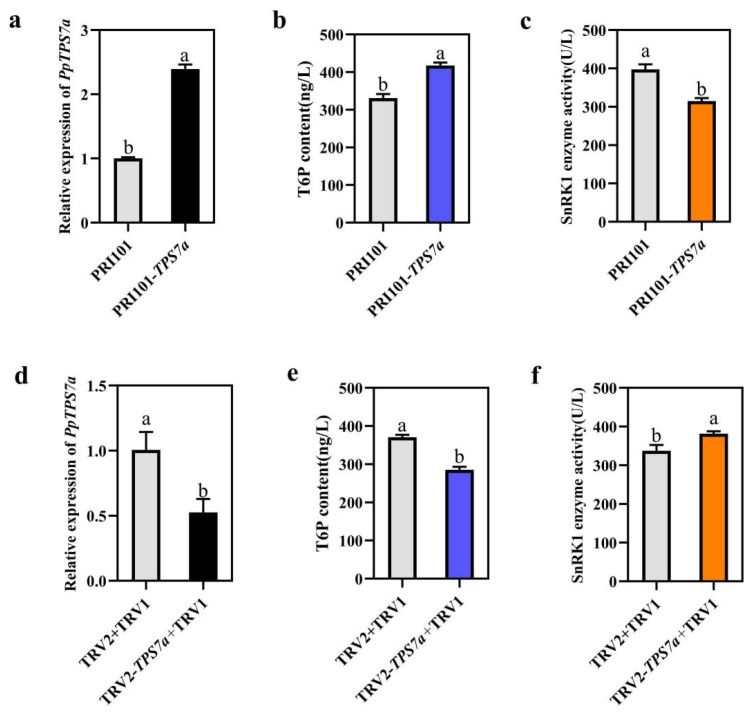
Gene function of *PpTPS7a*. (**a**) Relative expression level of *PpTPS7a* in transient overexpression peach fruit. (**b**) T6P content after transient overexpression of *PpTPS7a*. (**c**) SnRK1 activity after transiently overexpression of *PpTPS7a*. (**d**) Relative expression level of *PpTPS7a* in transient silencing peach fruit. (**e**) T6P content after silencing. (**f**) SnRK1 activity after silencing.

To validate the function of *PpTPS7a*, transient overexpression and gene silencing were performed in peach fruit. Overexpression of the *PpTPS7a* gene could significantly increase the T6P content (Figure 8a,b). while silencing *PpTPS7a* resulted in a decrease in T6P (Figure 8d,e). In addition, the enzyme activity of SnRK1 was also affected in transient expression fruit, showing decreased activity in overexpression fruit and increased silencing (Figure 8c,f). In conclusion, the *PpTPS7a* gene was mainly expressed in fruits and played a function in T6P synthesis during peach fruit development, which further regulated SnRK1 protein kinase activity.

## 4. Discussion

TPS is a central enzyme in trehalose metabolism, which is essential for plant growth and stress tolerance [40]. In this study, nine *TPS* genes were identified in the peach genome. *TPS* genes have been identified in many plant species, such as *A. thaliana* (*AtTPS1*–*AtTPS11*) [10,37], *Oryza sativa* (*OsTPS1*–*OsTPS11*) [11], and *Populus trichocarpa* (*PtTPS1*–*PtTPS12*) [9]. Compared to Arabidopsis, rice, and populus, there were fewer *TPS* genes in peaches, which suggested that *TPS* genes were amplified to different extents in different species. Phylogenetic analyses showed that *PpTPS* genes were mainly grouped into two subfamilies (Figure 3b), in agreement with *TPS* genes in other plants [41]. Multiple sequence comparisons showed that the average amino acid sequence concordance within the domains of TPS and TPP was high, while the amino acid sequences outside the domains varied widely (Figure 3a). These non-conserved regions may contribute to the functional differences in *TPS* genes.

The *PpTPS* gene structure showed that the number of introns and exons in class I was much higher than that in class II. In agreement with the findings of other studies, *TPS* genes in classes I and II have experienced different selective pressures, and *TPS* genes in class II have lost some introns during evolution due to strong selective pressures [42]. It has been shown that the apparent differences in expression patterns and functions between classes I and II might be related to extensive variation in exon and intron structure [43]. Further research is needed to explore the evolutionary origins of *TPS* gene introns and their impact on TPS protein function. To further investigate the evolutionary mechanism of *PpTPS* genes, collinearity analyses of *TPS* genes in peach and Arabidopsis were performed. A total of 11 *TPS* genes in Arabidopsis were matched with peach, while only three pairs of genes were found to be covariant in peach. This result indicated that *TPS* genes had high conservation. As well, in cotton, *TPS* family members seem to experience strong negative selection, which implies that these *TPS* were functionally conservative [44].

Numerous response elements were revealed in this study, including those in response to environment (light, anaerobic, low temperature) and plant hormones (IAA, GA, SA, ABA, and MeJA). The presence of hormone response elements (ABA, GA, SA, IAA) and environment response elements (light, anaerobic, low temperature) indicated that *PpTPS* genes might be involved in plant hormone and stress resistance signaling pathways, which was consistent with previous studies [45,46]. For example, overexpression of *AtTPS1* in Arabidopsis significantly improves drought tolerance [47]. Overexpression of *OsTPS8* in rice improves salt stress tolerance [18]. *OsTPS1* increases rice seed resistance to cold, salt, and drought [14]. While the promoters of *PpTPS1a* and *PpTPS1b* contain ABA, a low-temperature-responsive element (Figure 5a), we hypothesize that when peach trees are stimulated by changes in the external environment, the expression of *PpTPS1a* and *PpTPS1b* would be regulated, affecting T6P levels to maintain homeostasis in vivo. In this study, the light-responsive element (G-box) and the abscisic acid-responsive element (ABRE) are the two major cis-acting elements of the *PpTPS* family, as demonstrated in other studies. For example, light quality affects tomato growth by influencing the *TPS1*-T6P signaling pathway [48], and the ABRE element in the *TPS* gene promoter might participate in the response to ABA [16].

Tissue-specific expression patterns of *PpTPS* may explain their important role in specific tissues. *PpTPS1a* and *PpTPS1b* were specifically expressed in leaves, showing similar expression patterns. Two class I *TPS* genes also showed similar tissue expression patterns in watermelon [49]. *PpTPS5*, *PpTPS9a*, and *PpTPS9b* were highly expressed in stems, which may mediate stem development, *PpTPS6* and *PpTPS7a* were highly expressed in fruits; which might play an important role in fruit development. The function of class II *TPS* genes remains to be explored.

The gene expression pattern of *PpTPS* genes was analyzed during peach fruit development. The expression patterns of class I *TPS* genes were consistent and gradually decreased during fruit development, while the gene expression patterns were different in class II. As we know, T6P is a key sugar signaling molecule that further participates in carbohydrate metabolism by inhibiting SnRK1 [50,51]. In this study, we found that SnRK1 was completely opposite to the changes in T6P content during peach fruit development, which was consistent with previous studies. The correlation analysis between T6P and *TPS* expressions was carried out, showing that *PpTPS7a* has the highest correlation coefficient (R^2^ = 0.6208) (Figure S4 and Figure 7e). T6P is mainly synthesized by *TPS1* [52], while the expression of *PpTPS1a* and *PpTPS1b* during peach fruit development is not positively correlated with T6P content. Transient overexpression and gene silencing were performed in peach fruit, showing that the *PpTPS7a* gene played a role in T6P synthesis. T6P (trehalose-6-phosphate) is a plant compound that has a significant impact on metabolism, growth, and development [53]. *PpTPS7a* was shown to be significantly positively related to T6P concentration during fruit development, implying that it plays a role in peach fruit growth and development. The enzyme activity of SnRK1 was affected in transient expression fruit, showing decreased activity in overexpression *PpTPS7a* fruit and increased in silencing (Figure 8c,f). This result indicated that *PpTPS7a* might further participate in carbohydrate metabolism via SnRK1. In vitro kinase activity and cell tests have shown that SnRK1 is highly connected with class II T6P synthase (TPS)-like proteins, which regulate SnRK1 kinase activity and limit SnRK1 nuclear signal transmission [54]. *PpTPS7a* is a class II TPS protein with decreased SnRK1 activity in *PpTPS7a* overexpression fruit. *PpTPS7a* may interact with SnRK1 and inhibit its activity. Regulation of carbohydrate metabolism in peach fruit through the TPS-T6P-SnRK1 pathway.

Our study revealed a new mechanism for the regulation of T6P levels in peach fruit, which not only extended the function of *TPS* genes in class II but also provided a theoretical basis for carbohydrate regulation in peach fruit.

## 5. Conclusions

In summary, we identified nine *PpTPS* genes from peach and analyzed their conserved protein motifs, gene structure, chromosome distribution, cis-acting elements in the promoter region, and molecular evolution. The identified *TPS* genes and their potential function in regulating T6P-SnRK1 signaling provided insights into carbohydrate metabolism in peach fruit.

## Figures and Tables

**Table 1:** Physical and chemical properties of *TPS* gene family members in peach.

Name	Gene Accession	aa	Chrom	Chr-Start	Chr-End	MW (Da)	pI	GRAVY
PpTPS1a	Prupe.4G071400	926	Chr.4	3497894	3506676	104,186.14	6.88	−0.285
PpTPS1b	Prupe.4G155900	924	Chr.4	8926783	8938050	104,373.73	6.37	−0.378
PpTPS5	Prupe.5G031500	858	Chr.5	3611189	3618103	97,218.49	5.67	−0.19
PpTPS6	Prupe.1G334900	855	Chr.1	31647574	31652271	96,726.25	5.9	−0.19
PpTPS7a	Prupe.3G113100	840	Chr.3	9435987	9440491	95,686.72	6.19	−0.244
PpTPS7b	Prupe.4G067300	854	Chr.4	3258754	3263493	96,553.11	5.8	−0.267
PpTPS9a	Prupe.5G176400	862	Chr.5	14959038	14964334	97,797.31	6.39	−0.185
PpTPS9b	Prupe.1G095500	859	Chr.1	7551683	7555029	97,549.83	6.25	−0.207
PpTPS10	Prupe.1G256200	861	Chr.1	26652471	26657279	97,224.26	5.88	−0.24

Note: aa indicates amino acid length, MW indicates molecular weight, pI indicates theoretical isoelectric point, GRAVY indicates grand average of hydropathicity multiple sequence alignment.

## Data Availability

Data are contained in the article and Supplementary Materials.

## Supplementary Materials

The following supporting information can be downloaded at: https://www.mdpi.com/article/10.3390/genes15010039/s1, Table S1: The primer sequences of *PpTPS* genes for qRT-PCR and gene cloning; Figure S1: Comparison of TPS family full-length amino acids and TPS domains; Figure S2: Comparison of the TPS family’s TPP domains and amino acid sequences outside of the domain; Figure S3: Multiple sequence comparison of *TPS*; Figure S4: Correlation investigation of *TPS* expression and T6P content change during fruit development.
