# Supplementary material for: Genome-Wide Identification of Trehalose-6-phosphate Synthase (TPS) Gene Family Reveals the Potential Role in Carbohydrate Metabolism in Peach
Source: Genes (Basel). 2023 Dec 26;15(1):39. doi: 10.3390/genes15010039 (PMC10815152; doi:10.3390/genes15010039)
Supplement: Supplementary file 1 [file genes-15-00039-s001.zip › genes-2785643-supplementary.pdf]

Table S1 The primers sequences of *PpTPS* genes for qRT-PCR and gene cloning.

Gene	Primer-F	Primer-R
PpTPS1a	CATCTGTGGACAGGCCCAAT	TAACACCAACTGCTCGGACC
PpTPS1b	CATCTGTGGACAGGCCCAAT	TAACACCAACTGCTCGGACC
PpTPS5	ATGCCGCCAAGGAAATGAGA	AGCATCAATGTTCCACGGGT
PpTPS6	ACAAGTTCCATAGCAGGCCC	CTGCTCTGACACAGATGCCA
PpTPS7a	AAGTCCCCCAGTCAGAAGGT	TACCCATGTTCTGCCGCAAT
PpTPS7b	ATCATGGTGGGCGGTTTGAT	CAAGAAGGTAGGCAGCACCA

PpTPS9a	CCGACGACAACCACCAAAAC	CCAGGCTGTCATGGTCCAAT
PpTPS9b	TGTGAGGGATGGCATGAACC	TACGAGGGGAATCAGAGGCA
PpTPS10	TCTGAGCGATTGGTTTGCCT	TTCCAATCCAGATCGGCACC
Actin	GTTATTCTTCATCGGCGTCTTCG	CTTCACCATTCCAGTTCCATTGTC
PRI101-PpTPS7a	gaccccgggggtaccggatccATGTCGAAGTCATACGT AAATCTTTTAGA	ttctcctttacccatgaattcAAGGCTTTCAGAAAGAC ATTTAAGC
TRV2-PpTPS7b	gtgagtaaggttaccgaattcATCTATAGCACTCTTACT GTAAGAGAAGAAAT	cgtgagctcggtaccggatccTTCCATAAAATTCCAA TCCTAAGTAACC

a

Sequence 1:	PpTPS1b	926 aa
Sequence 2:	PpTPS9a	859 aa
Sequence 3:	PpTPS10	861 aa
Sequence 4:	PpTPS6	855 aa
Sequence 5:	PpTPS7b	840 aa
Sequence 6:	PpTPS7a	854 aa
Sequence 7:	PpTPS1a	924 aa
Sequence 8:	PpTPS5	858 aa
Sequence 9:	PpTPS9b	862 aa

Sequences (1:2)	Aligned.	Score: 26.7753
Sequences (1:3)	Aligned.	Score: 26.597
Sequences (1:4)	Aligned.	Score: 20.5848
Sequences (1:5)	Aligned.	Score: 25.8333
Sequences (1:6)	Aligned.	Score: 25.4098
Sequences (1:7)	Aligned.	Score: 76.9481
Sequences (1:8)	Aligned.	Score: 27.8555
Sequences (1:9)	Aligned.	Score: 26.7981
Sequences (2:3)	Aligned.	Score: 65.5413
Sequences (2:4)	Aligned.	Score: 61.5205
Sequences (2:5)	Aligned.	Score: 57.1429
Sequences (2:6)	Aligned.	Score: 60.6557
Sequences (2:7)	Aligned.	Score: 26.3097
Sequences (2:8)	Aligned.	Score: 61.8881
Sequences (2:9)	Aligned.	Score: 63.4459
Sequences (3:4)	Aligned.	Score: 64.5614
Sequences (3:5)	Aligned.	Score: 61.4286
Sequences (3:6)	Aligned.	Score: 63.8173
Sequences (3:7)	Aligned.	Score: 27.0616
Sequences (3:8)	Aligned.	Score: 64.4522
Sequences (3:9)	Aligned.	Score: 75.2613
Sequences (4:5)	Aligned.	Score: 62.619
Sequences (4:6)	Aligned.	Score: 65.6909
Sequences (4:7)	Aligned.	Score: 21.4035
Sequences (4:8)	Aligned.	Score: 74.9708
Sequences (4:9)	Aligned.	Score: 63.1579
Sequences (5:6)	Aligned.	Score: 77.2619
Sequences (5:7)	Aligned.	Score: 25.4762
Sequences (5:8)	Aligned.	Score: 64.6429
Sequences (5:9)	Aligned.	Score: 59.7619
Sequences (6:7)	Aligned.	Score: 25.9953
Sequences (6:8)	Aligned.	Score: 67.7986
Sequences (6:9)	Aligned.	Score: 62.7635
Sequences (7:8)	Aligned.	Score: 27.8555
Sequences (7:9)	Aligned.	Score: 27.1462
Sequences (8:9)	Aligned.	Score: 63.0536

b

Sequence 1:	PpTPS1a	485 aa
Sequence 2:	PpTPS1b	485 aa
Sequence 3:	PpTPS5	485 aa
Sequence 4:	PpTPS6	485 aa
Sequence 5:	PpTPS7a	485 aa
Sequence 6:	PpTPS7b	485 aa
Sequence 7:	PpTPS9a	485 aa
Sequence 8:	PpTPS9b	485 aa
Sequence 9:	PpTPS10	485 aa

Sequences (1:2)	Aligned.	Score: 84.9485
Sequences (1:3)	Aligned.	Score: 36.2887
Sequences (1:4)	Aligned.	Score: 35.4639
Sequences (1:5)	Aligned.	Score: 34.2268
Sequences (1:6)	Aligned.	Score: 33.4021
Sequences (1:7)	Aligned.	Score: 35.6701
Sequences (1:8)	Aligned.	Score: 34.433
Sequences (1:9)	Aligned.	Score: 34.0206
Sequences (2:3)	Aligned.	Score: 36.0825
Sequences (2:4)	Aligned.	Score: 36.2887
Sequences (2:5)	Aligned.	Score: 34.6392
Sequences (2:6)	Aligned.	Score: 33.1959
Sequences (2:7)	Aligned.	Score: 35.6701
Sequences (2:8)	Aligned.	Score: 34.0206
Sequences (2:9)	Aligned.	Score: 34.433
Sequences (3:4)	Aligned.	Score: 76.4948
Sequences (3:5)	Aligned.	Score: 67.4227
Sequences (3:6)	Aligned.	Score: 62.268
Sequences (3:7)	Aligned.	Score: 64.1237
Sequences (3:8)	Aligned.	Score: 63.5052
Sequences (3:9)	Aligned.	Score: 65.3608
Sequences (4:5)	Aligned.	Score: 66.3918
Sequences (4:6)	Aligned.	Score: 60.6186
Sequences (4:7)	Aligned.	Score: 64.9485
Sequences (4:8)	Aligned.	Score: 62.4742
Sequences (4:9)	Aligned.	Score: 64.9485
Sequences (5:6)	Aligned.	Score: 72.3711
Sequences (5:7)	Aligned.	Score: 61.2371
Sequences (5:8)	Aligned.	Score: 58.9691
Sequences (5:9)	Aligned.	Score: 61.2371
Sequences (6:7)	Aligned.	Score: 57.732
Sequences (6:8)	Aligned.	Score: 55.0515
Sequences (6:9)	Aligned.	Score: 57.9381
Sequences (7:8)	Aligned.	Score: 61.6495
Sequences (7:9)	Aligned.	Score: 74.6392
Sequences (8:9)	Aligned.	Score: 65.1546

Figure S1 Multiple sequence comparison of *TPS.* **a** Comparison of TPS family full-length amino acids. **b** Comparison of the TPS family's TPS domains.

a

```
Sequence format is Pearson
Sequence 1: PpTPS1a       113 aa
Sequence 2: PpTPS1b       113 aa
Sequence 3: PpTPS5        113 aa
Sequence 4: PpTPS6        113 aa
Sequence 5: PpTPS7a       113 aa
Sequence 6: PpTPS7b       113 aa
Sequence 7: PpTPS9a       113 aa
Sequence 8: PpTPS9b       113 aa
Sequence 9: PpTPS10       113 aa

Sequences (1:2) Aligned. Score: 67.2566
Sequences (1:3) Aligned. Score: 21.2389
Sequences (1:4) Aligned. Score: 23.0088
Sequences (1:5) Aligned. Score: 19.469
Sequences (1:6) Aligned. Score: 20.354
Sequences (1:7) Aligned. Score: 22.1239
Sequences (1:8) Aligned. Score: 20.354
Sequences (1:9) Aligned. Score: 25.6637
Sequences (2:3) Aligned. Score: 22.1239
Sequences (2:4) Aligned. Score: 20.354
Sequences (2:5) Aligned. Score: 20.354
Sequences (2:6) Aligned. Score: 21.2389
Sequences (2:7) Aligned. Score: 24.7788
Sequences (2:8) Aligned. Score: 24.7788
Sequences (2:9) Aligned. Score: 26.5487
Sequences (3:4) Aligned. Score: 49.5575
Sequences (3:5) Aligned. Score: 48.6726
Sequences (3:6) Aligned. Score: 46.9027
Sequences (3:7) Aligned. Score: 49.5575
Sequences (3:8) Aligned. Score: 46.0177
Sequences (3:9) Aligned. Score: 48.6726
Sequences (4:5) Aligned. Score: 46.9027
Sequences (4:6) Aligned. Score: 46.0177
Sequences (4:7) Aligned. Score: 47.7876
Sequences (4:8) Aligned. Score: 43.3628
Sequences (4:9) Aligned. Score: 45.1327
Sequences (5:6) Aligned. Score: 64.6018
Sequences (5:7) Aligned. Score: 55.7522
Sequences (5:8) Aligned. Score: 54.8673
Sequences (5:9) Aligned. Score: 54.8673
Sequences (6:7) Aligned. Score: 52.2124
Sequences (6:8) Aligned. Score: 52.2124
Sequences (6:9) Aligned. Score: 55.7522
Sequences (7:8) Aligned. Score: 53.9823
Sequences (7:9) Aligned. Score: 60.177
Sequences (8:9) Aligned. Score: 54.8673
```

b

```
Sequence format is Pearson
Sequence 1: PpTPS1a       95 aa
Sequence 2: PpTPS1b       95 aa
Sequence 3: PpTPS5        95 aa
Sequence 4: PpTPS6        95 aa
Sequence 5: PpTPS7a       95 aa
Sequence 6: PpTPS7b       95 aa
Sequence 7: PpTPS9a       95 aa
Sequence 8: PpTPS9b       95 aa
Sequence 9: PpTPS10       95 aa

Sequences (1:2) Aligned. Score: 43.1579
Sequences (1:3) Aligned. Score: 10.5263
Sequences (1:4) Aligned. Score: 10.5263
Sequences (1:5) Aligned. Score: 13.6842
Sequences (1:6) Aligned. Score: 9.47368
Sequences (1:7) Aligned. Score: 11.5789
Sequences (1:8) Aligned. Score: 8.42105
Sequences (1:9) Aligned. Score: 10.5263
Sequences (2:3) Aligned. Score: 11.5789
Sequences (2:4) Aligned. Score: 9.47368
Sequences (2:5) Aligned. Score: 11.5789
Sequences (2:6) Aligned. Score: 12.6316
Sequences (2:7) Aligned. Score: 11.5789
Sequences (2:8) Aligned. Score: 9.47368
Sequences (2:9) Aligned. Score: 9.47368
Sequences (3:4) Aligned. Score: 48.4211
Sequences (3:5) Aligned. Score: 60
Sequences (3:6) Aligned. Score: 53.6842
Sequences (3:7) Aligned. Score: 30.5263
Sequences (3:8) Aligned. Score: 34.7368
Sequences (3:9) Aligned. Score: 34.7368
Sequences (4:5) Aligned. Score: 51.5789
Sequences (4:6) Aligned. Score: 46.3158
Sequences (4:7) Aligned. Score: 31.5789
Sequences (4:8) Aligned. Score: 37.8947
Sequences (4:9) Aligned. Score: 37.8947
Sequences (5:6) Aligned. Score: 67.3684
Sequences (5:7) Aligned. Score: 34.7368
Sequences (5:8) Aligned. Score: 35.7895
Sequences (5:9) Aligned. Score: 41.0526
Sequences (6:7) Aligned. Score: 28.4211
Sequences (6:8) Aligned. Score: 28.4211
Sequences (6:9) Aligned. Score: 38.9474
Sequences (7:8) Aligned. Score: 32.6316
Sequences (7:9) Aligned. Score: 45.2632
Sequences (8:9) Aligned. Score: 34.7368
```

Figure S2 Multiple sequence comparison of *TPS*. **a** Comparison of the TPS family's TPP domains. **b** Comparison of amino acid sequences outside of the domain.

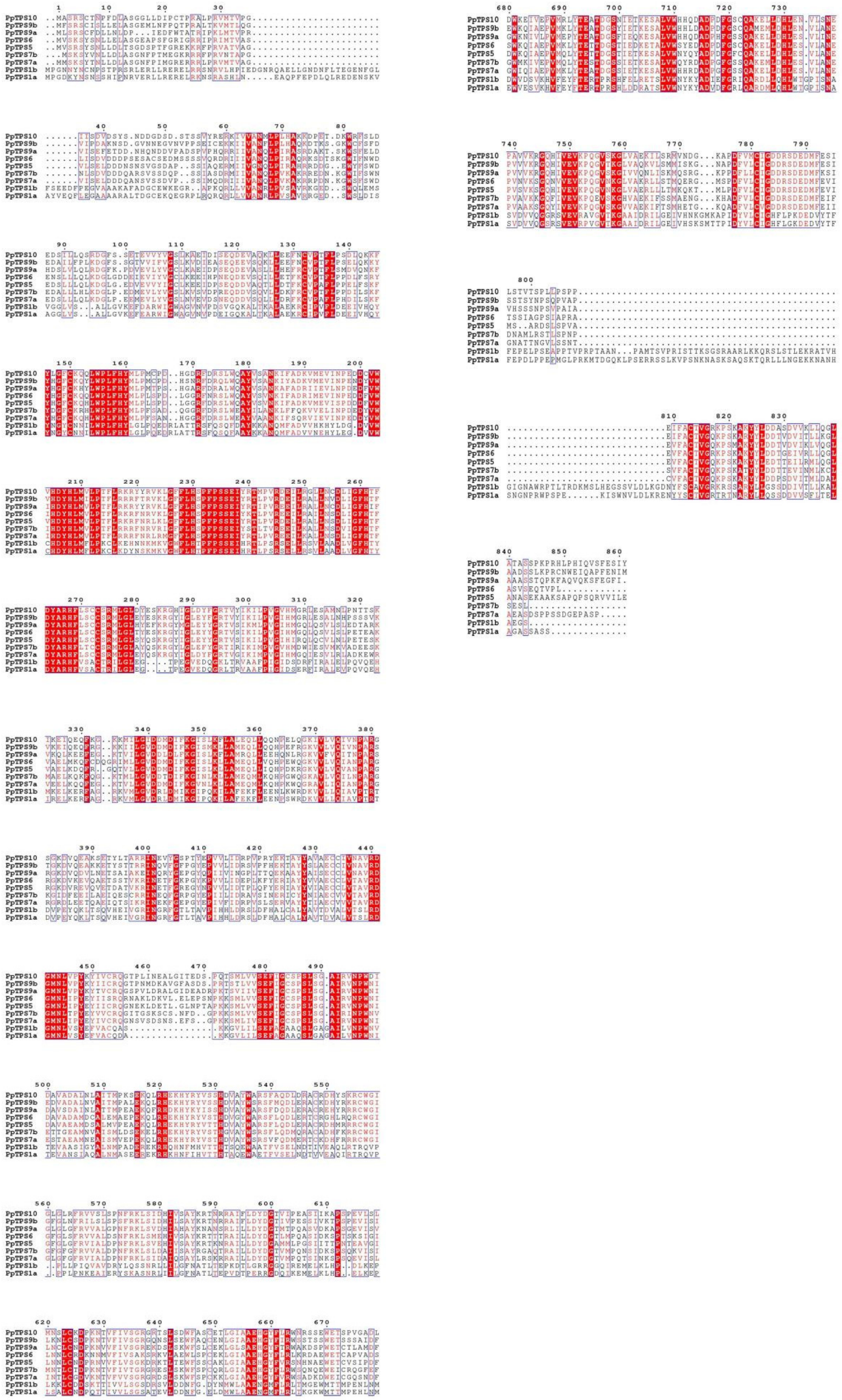

Figure S3 Multiple sequence comparison of *TPS*.

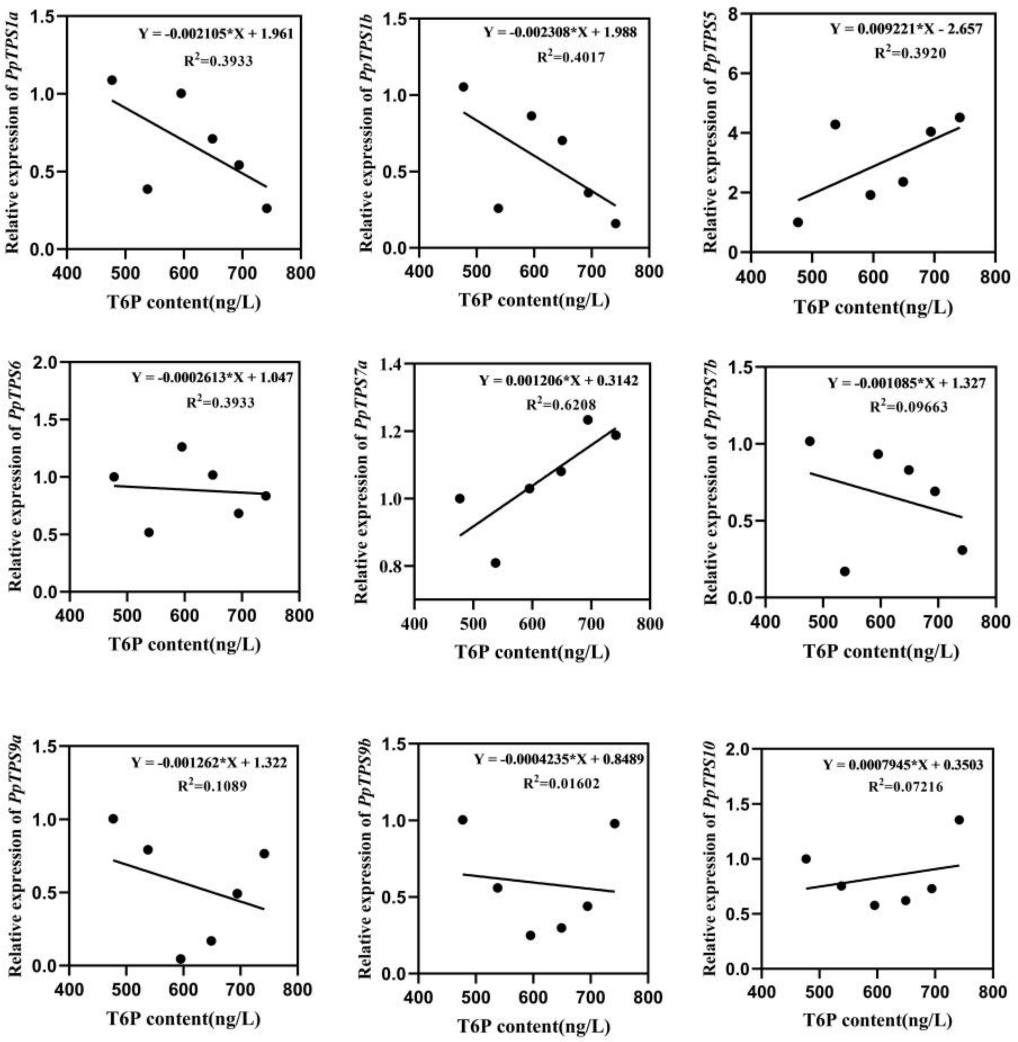

Figure S4 Correlation investigation of *TPS* expression and T6P content change during fruit development. The graph shows the regression equation and the R square.
